# Cisplatin Synergistically Enhances Antitumor Potency of Conditionally Replicating Adenovirus via p53 Dependent or Independent Pathways in Human Lung Carcinoma

**DOI:** 10.3390/ijms20051125

**Published:** 2019-03-05

**Authors:** Sakhawat Ali, Muhammad Tahir, Aamir Ali Khan, Xue Chai Chen, Ma Ling, Yinghui Huang

**Affiliations:** College of Life Science and Bioengineering, Beijing University of Technology, 100 Ping Le Yuan, Chaoyang, Beijing 100124, China; dr.sakhawatsaleem@hotmail.com (S.A.); m.tahir.qau@hotmail.com (M.T.); aamirkhan.uaar@outlook.com (A.A.K.); chenxuechai@bjut.edu.cn (X.C.C.); maling_rose@outlook.com (M.L.)

**Keywords:** cisplatin, chemotherapy resistance, apoptosis, lung cancer

## Abstract

Cisplatin is ranked as one of the most powerful and commonly prescribed anti-tumor chemotherapeutic agents which improve survival in many solid tumors including non-small cell lung cancer. However, the treatment of advanced lung cancer is restricted due to chemotherapy resistance. Here, we developed and investigated survivin promoter regulating conditionally replicating adenovirus (CRAd) for its anti-tumor potential alone or in combination with cisplatin in two lung cancer cells, H23, H2126, and their resistant cells, H23/CPR, H2126/CPR. To measure the expression of genes which regulate resistance, adenoviral transduction, metastasis, and apoptosis in cancer cells, RT-PCR and Western blotting were performed. The anti-tumor efficacy of the treatments was evaluated through flow cytometry, MTT and transwell assays. This study demonstrated that co-treatment with cisplatin and CRAd exerts synergistic anti-tumor effects on chemotherapy sensitive lung cancer cells and monotherapy of CRAd could be a practical approach to deal with chemotherapy resistance. Combined treatment induced stronger apoptosis by suppressing the anti-apoptotic molecule Bcl-2, and reversed epithelial to mesenchymal transition. In conclusion, cisplatin synergistically increased the tumor-killing of CRAd by (1) increasing CRAd transduction via enhanced CAR expression and (2) increasing p53 dependent or independent apoptosis of lung cancer cell lines. Also, CRAd alone proved to be a very efficient anti-tumor agent in cancer cells resistant to cisplatin owing to upregulated CAR levels. In an exciting outcome, we have revealed novel therapeutic opportunities to exploit intrinsic and acquired resistance to enhance the therapeutic index of anti-tumor treatment in lung cancer.

## 1. Introduction

Lung cancer is accepted as the most prevalent and fatal malignancy worldwide. This is primarily owing to its fast advancement to metastatic stage IV before diagnosis, particularly the non-small cell lung cancer (NSCLC) [1] which accounts for nearly 95% of all lung cancers [2]. Ongoing lung cancer treatments face numerous challenges including but not limited to, complexity and variety of lung cancer subtypes, and acquired resistance [3]. This highlights the significance of searching novel therapeutic strategies which not only can alleviate the current problems faced by clinicians but also culminate in the development of effective and long-lasting individual or combined targeted anti-cancer warheads. Importantly, it can lead the way to personalized medicine, mainly in the therapy of resistant cancers [4,5].

The current treatment modalities; surgery, chemotherapy, and radiation, have shortcomings including but not limited to toxicity towards normal cells, recurrence, and resistance. Among chemotherapy drugs, cisplatin is the most frequently employed against a variety of cancers. However, the increasing occurrence of resistance to this platinum drug in recurrent tumors has largely precluded its clinical use as monotherapy. Resistance to this drug is attributed to several factors including the increase in DNA repair and drug inactivation, and altered cellular accumulation [6]. ATP binding cassette (ABC) family member, P-glycoprotein (P-gp/ABCB1), decreases the intracellular accumulation of several anticancer drugs and is the most extensively explored ABC family drug efflux pump [7,8]. The overexpression of P-gp/ABCB1 enables cancer cells to resist many currently available chemotherapeutics [9]. Still, the mechanisms underlying the cells resistance to platinum drugs are not fully understood, and their understanding may provide clues for therapeutic strategies to enhance the efficacy of chemotherapy drugs in advanced lung cancer or other malignancies.

The indispensability of a novel anti-cancer strategy has led the researchers to explore gene therapy, biotherapy, and immunotherapies. Compared to current regimens, immunotherapy inherits a great theoretical advantage; it is cancer selective, non-destructive, and manipulates the host’s immune surveillance. However, in the clinical settings, it faces many obstacles such as the lack of powerful cancer-specific stimulants and ideal administration routes, and the existence of defective host immune systems [10].

Oncolytic viruses (OVs) are therapeutically very useful due to their capability of selectively infecting and replicating in tumors while sparing healthy cells. The OVs destroy invaded tumors either by inducing direct oncolysis and triggering cancer-specific immune responses, or causing vasculature shutdown [11]. Survivin is a member of the inhibitor of the apoptosis protein family (IAP). Expression of this IAP member in cancer cells is very high, but on the contrary it is negligible in healthy body cells. Many studies have demonstrated survivin’s transcriptional regulation in tumors [12,13,14,15]. OVs whose replication is put under the control of the survivin promoter (CRAd) can take advantage of this vulnerability to target and kill cancer cells. Many investigations have reported a cell adhesion protein, Coxsackievirus and adenovirus receptor (CAR), with a variable expression in different tissues and mostly scarce expression on cancerous cells. Besides the necessity of other receptors for internalization, the infection ability of OVs also requires a high expression of CAR. Biological or chemical agents which could enhance CAR expression in tumor cells, can be employed to increase adenoviral infectivity [16,17,18,19].

Cisplatin synergizes with CRAd, and in our previous study we demonstrated that cisplatin resistance might also play a crucial role in the regulation of CAR expression. The enhanced CAR expression owing to cisplatin pre-treatment allowed greater virus infectivity and appeared responsible for the synergy between CRAd and cisplatin in lung cancer [20]. Also, CRAd-monotherapy proved very lethal against chemotherapy resistant cells. Keeping in mind the heterogeneity of lung cancer, the goal of the current interrogation was to examine and validate the toxicity of CRAd alone or combined with cisplatin using some additional cisplatin sensitive and resistant lung cancer cells. Besides, the anti-metastatic potential of CRAd and its effects on epithelial to mesenchymal transition (EMT) are explored in this study.

## 2. Results

### 2.1. Multidrug Resistance and CAR in Cisplatin Resistant Lung Cancer Cells

**Figure 1:**
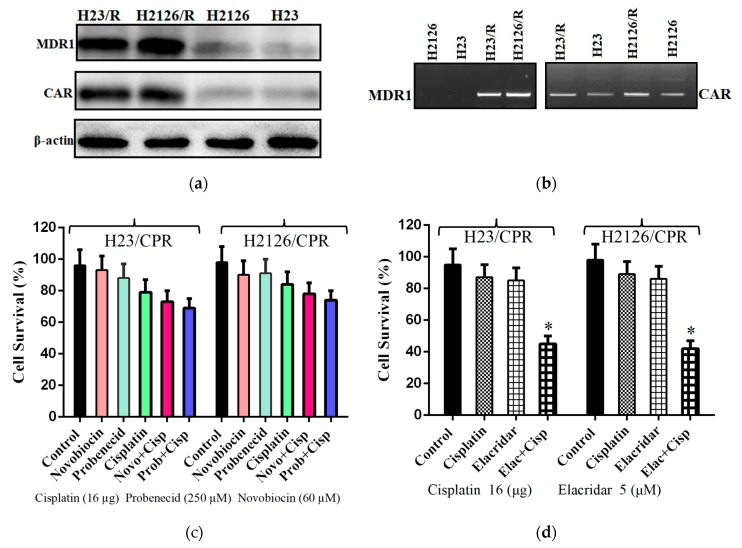
Cause of MDR phenotype in lung cancer cells. (**a**) Using anti-mdr1 antibody, Western blotting was performed. Results showed overexpression of MDR1 and CAR proteins in chemotherapeutic resistant cell lines of both lung cancer cells. Blot images were cropped for clarity of the presentation. (**b**) Reverse transcription PCR showing comparative mRNA levels of CAR and MDR1 in resistant and sensitive cells. (**c**) Cell viability assay to analyze the impact of Probenecid and Novobiocin treatments on resistance. (**d**) Sensitization of resistant cells to chemotherapy by elacridar treatment. Data are presented as the mean ± SD in (c) and (d), (*n* = 3), * *p* < 0.01, by two-tailed Student’s *t*-test.

Messenger RNA levels of ABCB1 (MDR1), ABCG2 (BCRP), ABCC1 (MRP1), and ABCC2 (MRP2) genes in lung cancer cells and their resistant sublines were evaluated by RT-PCR (Figure 1b). Subsequently, Western blotting was performed for genes with upregulated mRNA level. The results of these experiments showed that the MDR1 gene is responsible for resistance in H23/CPR and H2126/CPR sublines of lung cancer. Also, compared to sensitive cells, CAR was upregulated at both mRNA and protein levels in resistant cells (Figure 1a,b). Furthermore, Novobiocin (60 µM) which is a Breast Cancer Resistance Protein (BCRP/ABCG2) inhibitor [21] and Probenecid (250 µM), a multidrug resistance-associated protein (MRP-1) inhibitor [22] were also employed to exclude the chance of other causes of resistance. Cancer cells were first treated with these inhibitors and then exposed to cisplatin, but it had no significant increase in the cytotoxicity of the drug (Figure 1c). Also, a P-glycoprotein inhibitor (Elacridar) was employed to further confirm the cause of resistance. Resistant cells were first treated with elacridar (5 µM) and then exposed to 16 µg of cisplatin. Figure 1d shows that sensitivity of resistant cell lines to cisplatin was significantly increased when exposed to elacridar. These results suggest that the MDR phenotype in cancer cells was caused by the overexpression of ABCB1/MDR1 gene and not BCRP, MRP-1 genes.

### 2.2. Cisplatin-Resistant Lung Cancer Cells are More Sensitive to Adenoviral Infection with CRAd

**Figure 2:**
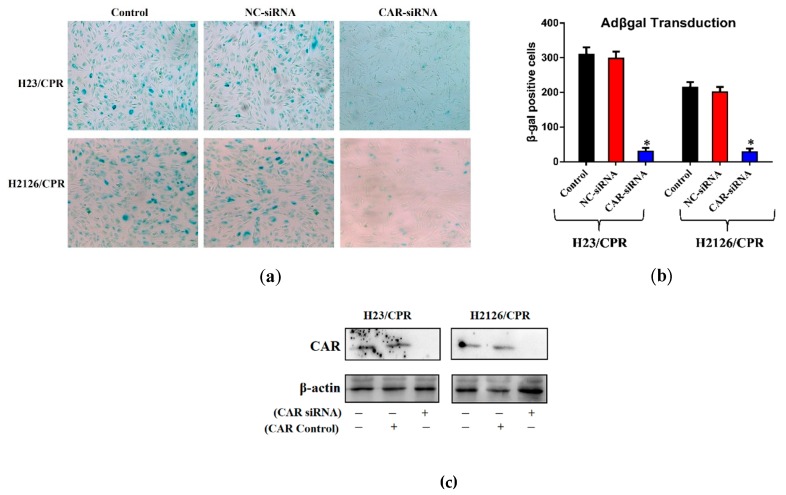
CAR knockdown and adenoviral transduction efficacy. (**a**) Both lung cancer cell lines were evaluated for CRAd infection efficacy through X-gal staining. Lung cells were cultured in 6-well plates and treated with different Adβgal doses. After 48 h, all cells were washed, fixed, stained and photographed via phase contrast microscope 100× magnification. (**b**) Resistant cells exhibit higher rates of transduction and siRNA medicated CAR knockdown significantly reduces the infectivity. (**c**) Western blots showing CAR overexpression in resistant sublines of lung cancer cells and CAR knockdown by employing CAR siRNA. Data are presented in (**b**) as the mean ± SD, (*n* = 3), * *p* < 0.01, by two-tailed Student’s *t*-test.

**Figure 3:**
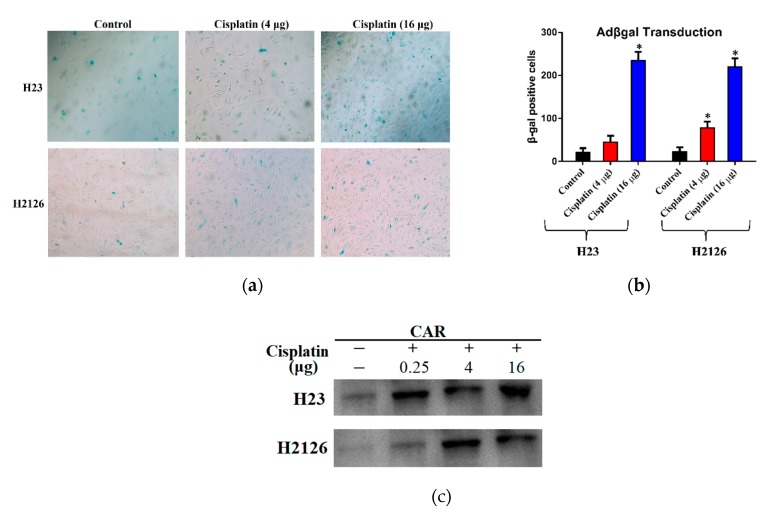
Effect of cisplatin treatment on CAR levels and adenoviral transduction. (**a**) Lung cancer cell lines, H23 and H2126, were treated with different cisplatin doses and observed for CRAd infection efficacy through X-gal staining. Cells were cultured in 6-well plates and treated with different Adβgal doses. After 48 h, all cells were washed, fixed, and stained and observed under the phase-contrast microscope (100× magnification). (**b**) Cells exhibit higher rates of transduction at high doses of cisplatin. (**c**) Western blots showing CAR overexpression in cancer cell lines pre-treated with cisplatin. Data are presented in (**b**) as the mean ± SD, (*n* = 3), * *p* < 0.01, by two-tailed Student’s *t*-test.

To confirm whether the increased transduction efficiency of cisplatin-resistant cells could be the outcome of increased CAR protein on their surface, X-gal staining was performed. Protein levels of CAR in both cisplatin-resistant lung cancer sublines (H23/CPR, H2126/CPR) after CAR knockdown via siRNA (CAR-siRNA) were compared with those of control cells (negative control, NC-siRNA), using Western blotting. The results show that siRNA successfully knockdown the CAR in targeted cancer cells (Figure 2c). Furthermore, the impact of CAR knockdown on transduction efficiency of adenovirus was evaluated through X-gal staining experiments. Results showed that CAR knockdown in both cisplatin-resistant cancer sublines substantially decreases adenoviral transduction (Figure 2a). Control cells exhibited a many fold higher number of βgal-positive cells than the cells which received CAR siRNA (Figure 2b). Figure 2b show that cancer cells which received NC-siRNA were infected 5–10 fold more efficiently compared to CAR-siRNA receiving cells. Hence, this significant difference in transduction efficacy between the two groups could be due to increased CAR expression. These results are in agreement with previous studies [23]. The experiments suggest that the success of adenovirus-based strategies is associated with CAR expression which is generally low at cancer cells Figure 1a,b) but could be high in chemotherapy-resistant tumors and in cases where cells are pre-treated with cisplatin (Figure 1a, Figure 2c and Figure 3c).

### 2.3. Cisplatin-Mediated CAR Upregulation is Responsible for Raised Transduction Insensitive Cells

To determine the susceptibility of cisplatin-sensitive cancer cell lines to adenoviral infection, we opted for the same procedure as above. Cell lines (H23, H2126) were first treated with varying doses of cisplatin (4 μg or 16 μg) or only DMSO (control). Though the respective infectability of both cell lines indicated by X-gal staining was variable, a significant difference was observed between the control and cisplatin-treated cells (Figure 3a). As compared to the control, the number of βgal-positive cells were increased 2–3 fold with a cisplatin dose of 4 μg/mL, and more than 5-fold increase was observed at 16 μg/mL. The result of X-gal staining exhibits a dose-dependent increase in transduction efficiency (Figure 3b). Western blots reveal that the mechanism behind increased adenovirus infectivity was cisplatin-mediated CAR upregulation (Figure 3c).

### 2.4. Synergistic Antitumor Effect of Cisplatin and CRAd in Cancer Cells

**Figure 4:**
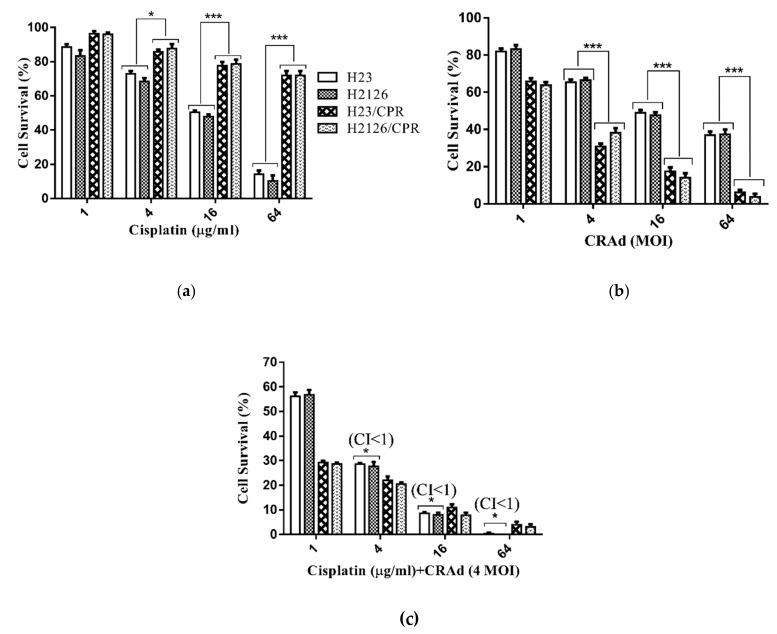
Tumor cell viability analysis. (**a**) Cisplatin decreases the viability of chemo-sensitive lung cancer cell lines, H23 and H2126, in a dose-dependent manner but resistant sublines give a poor response. (**b**) Both lung cancer cell lines were infected with variable MOIs (1–64) of CRAd. Cisplatin-resistant cells exhibit remarkably high tumor inhibition as compared to sensitive cells. (**c**) Co-treatment of cisplatin with CRAd results in very high initial inhibition rate even at a lower dose of cisplatin, and a continuous increase in inhibition was seen with increasing cisplatin doses. Cisplatin-sensitive cells showed a decrease in the cancer cell population in a synergistic manner (CI < 1). To calculate CI values following the Chou–Talalay method, CompuSyn 2.0 software was used. The data shown are the average of triplicate experiments. Data are presented as the mean ± SD in (**a**–**c**), (*n* = 3), * *p* < 0.05, *** *p* < 0.001, by two-tailed Student’s *t*-test.

Cell viability assay revealed that cisplatin monotherapy was effective against cisplatin-sensitive cells and the tumor cell killing response was enhanced as the dose increased from 1 μg to 64 μg. There was a prominent difference among the cell viabilities of cisplatin-sensitive and resistant cell lines at respective doses. Maximum cancer cell inhibition (≥85%) was achieved in cisplatin-sensitive cells at 64 μg/mL dose while only 25–30% inhibition was observed in resistant cells at the same dose (Figure 4a). A nearly reverse trend in cancer cell viabilities was found when cisplatin-sensitive and resistant lung cancer cells were infected with CRAd. Although in both groups, cell survival was decreased in a concentration-dependent manner, the effect was more pronounced in cisplatin-resistant cells (H23/CPR, H2126/CPR) as compared to that of sensitive cells. At multiplicity of infection (MOI) 4, the percent cell viability of sensitive cells was above 65%, but in resistant cells, it was reduced to 35%. Similarly, at MOI 16, it was as low as 15% in resistant cells as compared to nearly 50% of cisplatin-sensitive cells (Figure 4b). This significant difference in cell viabilities upon CRAd treatment was due to very high CRAd transduction and subsequent oncolysis owing to raised CAR expression in cisplatin-resistant cells.

Co-treatment of cisplatin and CRAd synergistically reduces cancer cell viability in cisplatin-sensitive lung cancer cells (H23, H2126). To leave the window open for any noticeable synergistic or additive effects of combined treatment, we selected MOI 4 of CRAd dose. All cancer cells were first treated with cisplatin (1 μg/mL to 64 μg/mL), and then after 10–12 h infected with CRAd. Results of the MTT assay displayed that combined treatment responded in a synergistic (CI < 1) manner in cisplatin-sensitive cancer cells. Although both cisplatin-resistant cells showed no synergy (CI ≥ 1), the reduction in percent cell viability was comparable to that of the combined treatment effects on cisplatin-sensitive cells (Figure 4c). These results are similar to those obtained in our previous study with lung cancer cells (A-549 and A-549/DDPR) [24].

### 2.5. Cisplatin and CRAd Inhibited Cancer Cell Migration

**Figure 5:**
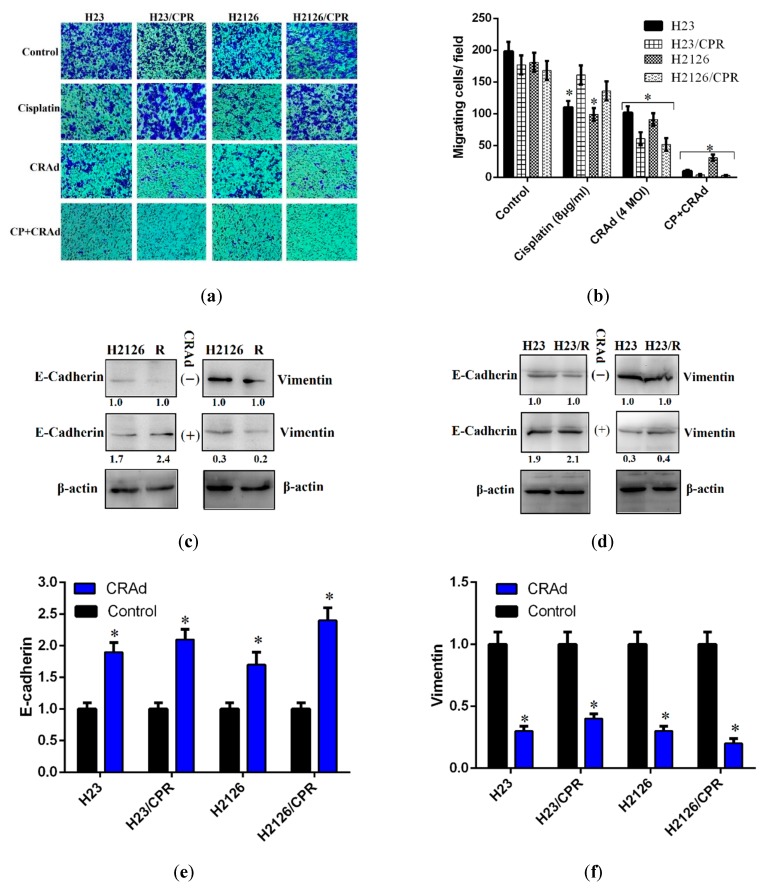
Tumor cell migration study via transwell assay: (**a**) Micrographs taken through a phase contrast microscope (100× magnification) showing migrated cancer cells in three treatment groups and DMSO control. (**b**) Results show that CRAd monotherapy reduces the migrating cells to 100 as compared to 200 migratory cells in the control. Cisplatin monotherapy also inhibits the migration of cancer cells. However, the combination therapy exerts the most potent inhibition on cell migration. Data are presented as the mean ± SD (*n* = 3), * *p* < 0.01, by two-tailed Student’s *t*-test. (**c**–**f**) Western blotting was performed to evaluate the expression of epithelial to mesenchymal transition markers. Total cell lysates were extracted and blotted with anti-E-cadherin, anti-vimentin antibodies, and resulting bands were normalized to the level of β-actin, and intensities were quantified relative to the untreated control as shown. Results show that compared to controls, protein levels of E-cadherin were increased while that of vimentin were decreased when cancer cells were treated with CRAd (* *p* < 0.01). The data shown above are the average of triplicate experiments.

Metastasis is a prominent factor in cancer-related deaths. Although inhibition of cancer cells proliferation is very crucial, the cells potential to migrate is also of equal concern for the cancer metastasis. We investigated the anti-migratory potential of cisplatin and CRAd monotherapies, and their combined therapy (4 μg cisplatin + 2 MOI CRAd). Transwell assay (Boyden chamber assay) was carried out for cisplatin-resistant and sensitive lung cancer cells, and the results of treatment groups are displayed in comparison to the DMSO control. The micrographs demonstrated that resistant cells exposed to cisplatin or DMSO migrate at comparable rates towards the chemoattractant (DMEM + 10% FBS) in the lower chamber of the millicells. Monotherapies of both cisplatin and CRAd inhibit migration, but the difference was more prominent among sensitive and resistant cells when treated with CRAd (Figure 5a). The number of cisplatin-resistant cells migrating per field when treated with CRAd (MOI 4) was nearly 50 compared to 100 in the case of cisplatin-sensitive cells (Figure 5b). The combined treatment group exhibits the most potent inhibition of migration as the number of migrating cells per field remained below 10 in cancer cell lines except H2126 where migrating cells were 30 (Figure 5b).

Different studies have highlighted the significant role of EMT-markers in metastasis of tumors. CRAd monotherapy was very successful in reversing EMT which reduces the metastatic potential of cancer cells. To explore the mechanism behind this, we performed RT-PCR and Western blot analysis for the EMT-markers, E-cadherin, and vimentin. Results of this investigation indicated that in CRAd treated cells, protein levels of E-cadherin were relatively upregulated while that of vimentin were downregulated. The lung cancer cells which did not receive any treatment showed nearly the opposite trend (Figure 5c–f). These results are consistent with those reported by Yuuri Hashimoto [25] and needs further investigation.

### 2.6. Cisplatin and CRAd Induce Apoptosis in Lung Cancer Cells by Activating the Caspase Pathway

**Figure 6:**
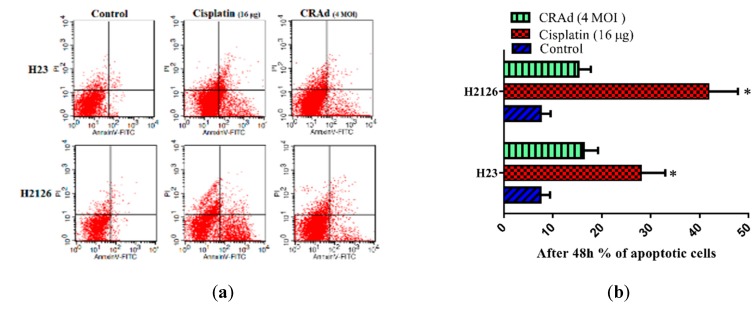

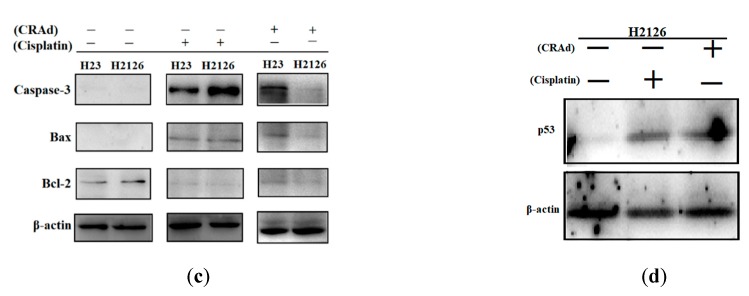
Effects of monotherapies of cisplatin and CRAd on apoptosis in lung adenocarcinoma cells. (**a**,**b**) Flow cytometry was performed to evaluate the impact of treatments on apoptosis. Results showed that both cisplatin and CRAd increases apoptosis in H23 and H2126 lung cancer cells as compared to DMSO treated controls. One out of three of the experiments with the same results is shown (* *p* < 0.01). (**c**) Western blots showed that the protein levels of bax and caspase-3 are increased while that of bcl-2 (anti-apoptotic protein) is reduced. It suggests that both treatments activate mitochondria/caspase apoptotic mechanism. (**d**) Similarly, p53 expression was also observed to be increased in H2126 lung cancer cells in both treatments groups.

Apoptosis is a category of programmed cell death and is controlled by the homeostatic balance between pro-apoptotic and anti-apoptotic genes. Dysregulation of these genes in cancer cells causes a decrease in cell death (apoptosis). To determine the impact of cisplatin and CRAd therapies on apoptosis, and to reveal the molecular mechanisms responsible for any change in cancer cells apoptosis status, we performed flow cytometry (FACS) and Western blotting. Figure 6a,b shows that compared to untreated controls, the number of apoptotic cells determined through the FACSCalibur system after treating lung cancer cells with cisplatin or CRAd for 48 h is markedly increased. Cisplatin (16 μg/mL) induces stronger apoptosis than CRAd infection at MOI 4. At 16 μg/mL of cisplatin dose, a significant increase in total apoptosis was observed in both H23 lung cancer cells (28% apoptosis) and H2126 cells (42%). CRAd treatment (MOI 4) nearly doubles apoptotic cells percentage (15–16%) in both lung cancer cells as compared to control (Figure 6b).

Protein level analysis via Western blotting shows that in lung cancer cells treated with cisplatin or CRAd, the level of anti-apoptotic bcl-2 was reduced while pro-apoptotic bax and caspase-3 levels were enhanced (Figure 6c). These molecular changes might have triggered the mitochondria/caspase pathway of apoptosis. Furthermore, the increase in p53 protein level was also observed in both treatment groups (cisplatin, CRAd) but only in H2126 lung cancer cells (Figure 6d). Hence, cisplatin in chemo-sensitive (MDR-) cells significantly enhanced caspase-3 activities. Also, it markedly increased the protein levels of bax and p53 (H2126 cells) and decreased the expression of Bcl-2, which ultimately led to a significant increase in cancer cell death. Based on these results, we can assume that activation of the intrinsic pathway causes cisplatin and CRAd-induced apoptosis. We conclude that both cisplatin and CRAd could elicit the mitochondria/caspase apoptotic mechanism in cisplatin-sensitive lung cancer cells.

### 2.7. Co-Treatment of Cisplatin with CRAd Promotes Apoptosis Dependent and Independent Death of Lung Adenocarcinoma Cells in a Synergistic Manner

**Figure 7:**
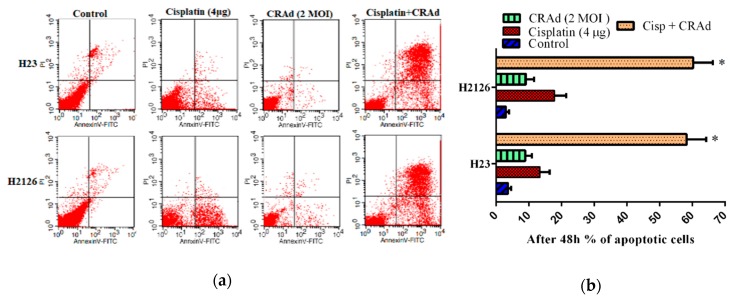
The apoptotic potential of co-treatment of cisplatin with CRAd. (**a**,**b**) Flow cytometry was performed to evaluate the impact of combined treatment on apoptosis. Lung cancer cells were first exposed to a reduced dose of cisplatin (4 μg/mL), and after 6–8 h infected with CRAd (MOI 2). Synergistic apoptotic death was achieved by combined treatment. One out of the three experiments with the same results is shown. (* *p* < 0.01).

Co-treatment of cisplatin and CRAd enhanced total cancer cell death to a very great extent as shown in cell viability assay. To evaluate how this strategy influences the level of apoptosis, we performed flow cytometry for the combined treatment similar to that for our monotherapy treatments. The doses of cisplatin and CRAd were reduced as compared to those used in monotherapy. The doses were reduced to highlight the potential of combined treatment strategy. In sequential treatment, lung cancer cells were first exposed to a reduced dose of cisplatin (4 μg/mL), and after 6–8 h infected with CRAd (MOI 2). Results illustrated in Figure 7a,b show that overall cell death was increased synergistically (Cl <1). In conclusion, the cisplatin synergistically increased the tumor-killing of CRAd by (1) increasing CRAd transduction via enhanced CAR expression and (2) increasing p53 dependent or independent apoptosis of lung cancer cell lines.

## 3. Discussion

Cisplatin is ranked as one of the most powerful and commonly prescribed anti-tumor chemotherapeutic agents which improved survival in many solid tumors including NSCLC [26,27,28]. It interacts and forms adducts with DNA which leads to the activation of some signal transduction pathways (MAPK, p53) resulting in apoptotic death of the injured cell. Although a platinum-based combination strategy is the benchmark for NSCLC, response rates are generally below 40% because of intrinsic resistance. Response to this strategy is further lowered to 10–20% in the case of acquired resistance [29,30].

Use of replication-competent viruses in oncolytic virotherapy to selectively destroy malignancies is a promising therapeutic strategy. However, their therapeutic effects are dependent on the CAR expression which is generally low on the cancer cell surfaces resulting in reduced viral entry [31,32]. Thus, to overcome this resistance to OVs, combination strategies should be designed. Here, through a variety of experiments, we showed that cisplatin sensitizes cancer cells to CRAd by upregulating CAR and synergistically improves therapeutic effects in lung cancer cells. Additionally, along with cisplatin, infection of CRAd also triggers the transcription of apoptotic cascade proteins (bax, bcl-2, caspase-3) resulting in enhanced tumor selective apoptotic killing.

In this study, we demonstrated that co-treatment of cisplatin with CRAd is a novel antineoplastic strategy with promising tumor-specific therapeutic efficacy. This approach could achieve the desired results at lower doses of chemotherapeutic agent. Also, it modifies signal transduction pathways in a way which synergistically enhances cancer cells death. Here, we employed two lung cancer cells, H23 and H2126, and their cisplatin resistant sublines, H23/CPR and H2126/CPR. We verified that the cisplatin-mediated upregulation of MDR1 (ABCB1) gene is responsible for resistance in H23/CPR and H2126/CPR sublines. Our results of X-gal staining experiments indicated that the presence of high levels of CAR on cancer cell surfaces are mandatory for effective CRAd infection. Furthermore, in cisplatin-resistant lung cancer cells, CAR upregulation seems a blessing in disguise culminating in very high CRAd transduction and subsequent oncolysis of these cells (Figure 2a–c).

Usually, NSCLC patients are already at an advanced stage when diagnosed, and clinicians are left with very few treatment options. Metastasis further worsens this condition leaving chemotherapy with a minimum efficacy to be used at advanced NSCLC stage. Cancer cells migration/invasion disseminates them throughout the body and hence is very crucial for metastasis. It renders cancer challenging to treat and is a prominent factor in cancer-related deaths. In the current study, we tested cisplatin and CRAd as monotherapies and in combination for their possible anti-metastatic potential. Results of transwell experiments indicated that CRAd inhibited cell migration in both chemo-sensitive and resistant lung cancer cells, and it seems that it does so by reversing EMT which is the hallmark of metastasis [33]. The combined treatment group involving both cisplatin and CRAd proved to be the most potent anti-migratory approach. These results are similar to a previous study which investigated the effects of oncolytic adenovirus in lung cancer cells induced with EMT [25] and needs further investigation.

Apoptosis (programmed cell death) and necroptosis, are barriers that impede malignant cells from proliferating and spreading in the host body. However, these natural barriers are evaded by cancer cells as they adopt various strategies via genetic mutations or other modifications in the modulators of these pathways [34]. Here, we performed flow cytometry and Western blotting to investigate the effect of cisplatin and CRAd treatments on programmed cell death and its molecular mechanism in lung cancer cells. The results showed that cisplatin effectively elicits apoptosis in sensitive cells by increasing the bax: bcl-2 ratio and enhancing expression of caspase-3 and p53 (protein level of p53 was increased only in H2126 lung cancer cells). Also, co-treatment of cisplatin with CRAd proved very useful in augmenting apoptosis in cancer cells.

In summary, this study revealed that the resistance to chemotherapy in lung adenocarcinoma could be treated with CRAd alone. Also, co-treatment of cisplatin with CRAd could synergistically cause tumor-specific killing in cancer cells sensitive to chemotherapy. These results might have important implications and we advocate that a strategy directed to both molecular targets, CAR and bax/bcl-2, could offer a promising therapeutic response to deal with lung adenocarcinoma and other tumors. Additional studies involving tumors of other origin as well as clinical trials are warranted to confirm the possible use of this innovative treatment approach in clinics and move it from bench to bedside.

## 4. Materials and Methods

### 4.1. Cell Culture and Reagents

Both human lung cancer cell lines (NCI-H23, and NCI-H2126) and HEK-293 cell line were procured from CAS-China and Microbix Biosystems Inc. respectively (Beijing, China). HEK-293 contains adenovirus (Adv) E1A-region for the replication of CRAd.

Cisplatin-resistant sublines, H23/CPR and H2126/CPR, were developed in our lab as described previously [24]. Afterward, all cancer cells were maintained in DMEM with 10% FBS and 2% of penicillin-streptomycin (HyCloneTM, Thermo Fisher Scientific, Waltham, MA USA USA). Doxorubicin (hydrochloride), cisplatin, elacridar, palbociclib, novobiocin, and probenecid were purchased from MedChemExpress (Shanghai, China) while puromycin was from Sigma-Aldrich. CRAd (incorporated with survivin promoter to regulate E1A region of Adv) was developed in our laboratory as described previously [35]. Ad-Luc expressing the firefly luciferase gene was used as a control.

### 4.2. Adv Transduction Assessment via β-Gal Activity

For transduction analysis, 10^6^ cancer cells were plated in 6-well plates and 24 h later infected with a varying multiplicity of infection (MOI) of Adβgal. 48 h post infection; cells were fixed using 0.5% glutaraldehyde and subsequently stained and analyzed under the light microscope for the expression of β-galactosidase. Blue-colored cells indicating transduction were counted. For culture, infection, fixation, and staining standard protocols (Beyotime) were followed.

### 4.3. siRNA and Transfection

Cisplatin-resistant lung cancer cells (H23/CPR and H2126/CPR) with confirmed CAR overexpression, were selected for siRNA mediated CAR silencing. For silencing of CAR, the predesigned human CAR-specific siRNA sequences and control siRNA (negative control, NC-siRNA) cloned into the ‘P-super’vector system were used (Santa Cruz Biotechnology, Inc, Dallas, USA). Following sequences were employed in siRNAs [36]: CAR-siRNA: CCAAGUACCAAGUGAAGACdTdT; NC-siRNA: CACAAAAGUAUCGCGCAAGdTdT.

For stable (puromycin-selection) transfection of siRNAs into the cancer cells, lipofectamine reagent was employed as per the manufacturer’s instructions (Santa Cruz Biotechnology, Inc, Dallas, USA). For all experiments, 40 nM siRNAs were used. The success of the silencing procedure was evaluated 48 h later with siRNA transfection through Western blotting.

### 4.4. Western Blotting

The expressions of MDR1 protein, CAR protein, and p53, bcl-2, bax, caspase-3 (apoptosis-related proteins), and E-cadherin, vimentin (EMT-markers) were analyzed by Western blotting. An amount of 10^6^ cancer cells of all cell lines were harvested for the total protein contents which were extracted using RIPA buffer (Beyotime Biotechnology, China), and quantized by employing BCA Protein Assay Kit (Beyotime Biotechnology, China). SDS-PAGE was done to separate proteins and subsequently transferred to PVDF membranes (Millipore, Billerica, MA) and blocked with nonfat dry milk overnight, and incubated with rabbit mAB. Antibodies were procured from Bioss and all treatments were carried out as per manufacturer’s protocol (Bioss Inc. USA). Finally, the proteins were visualized by chemiluminescence assay with an ECL kit. Images were used for semi-quantitative measurements based on band densitometry. The housekeeping gene β-actin was viewed as internal reference control [37].

### 4.5. Reverse Transcription-qPCR

cDNA was generated using the total RNA extracted (via TRIzol reagent) and purified from the cancer cells plated in 6-well plates. RT-PCR was carried out in triplicate by using a SYBR premix (TaKaRa Bio, Japan). The amplified products of ABCG2 (BCRP), ABCC1 (MRP1), and ABCC2 (MRP2) were observed via gel electrophoresis and the 2^−∆∆*C*t^ method. GAPDH was used as an endogenous control. The primer sequences used are listed in Table 1.

### 4.6. MTT Assay

The MTT assay was employed to determine cisplatin and CRAd in vitro dose response. All cancer cells were seeded in 96-well plates (10^4^/well), and 24 h later incubated with varying concentrations of cisplatin (1–64 µg/mL), and CRAd (1–64 MOI) alone or in combination. After 2–4 days of incubation, 10 μL/well of MTT (5 g/L) reagent was added, followed by further 4 h incubation at standard conditions (37 °C, 5% CO_2_). DMSO was used to dissolve formazan crystals, and finally, the absorbance was measured in a DNA microplate reader at 570 nm. The absorbance of control wells (CW) was taken as 100%, while in treated wells (TW) the percentage of the cells survival was determined using the given formula: (Absorbance of TW/Absorbance of CW) ×100 = Percent cell survival Percent cell survival was plotted against drug dose. These experiments were done in triplicates.

### 4.7. In Vitro Cell Migration Assay

The effect of cisplatin and CRAd on cancer cells migration was evaluated via a transwell assay [45,46]. The upper transwell inserts diluted with DMEM (no FBS) contained matrigel-coated membranes (BD Bioscience, San Jose, CA, USA), while the lower chamber contained chemoattractant (DMEM + FBS). An amount of 10^5^ cancer cells prepared in DMEM (no FBS) were plated onto upper 24-well millicell chambers, treated with CRAd, cisplatin or DMSO, and finally incubated at 37 °C in 5% CO2. After 8–12 h, cells which migrated to the lower chamber were fixed by applying 4% paraformaldehyde and stained for 15 min with crystal violet (0.5%) and subsequently washed with PBS. Phase contrast microscope (Leica, USA) was used to observe air dried migrated cells in the lower chamber, and their average number was counted by randomly choosing different views under the microscope. The same experimental procedure, but without chemoattractant, was performed for control (positive) groups. These experiments were done in triplicates.

### 4.8. Statistical Analysis

Each experiment was performed in triplicate, and to analyze the data obtained, GraphPad Prism6.0 was employed. To analyze data from experiments the two-tailed student’s *t*-test was applied. Statistical significance was set as * *p* < 0.05. ** *p* < 0.01, *** *p* < 0.001. Synergism of cisplatin and CRAd was analyzed, and CI values were calculated following the Chou–Talalay method using the CompuSyn 2.0 software (ComboSyn, Inc., Paramus, NJ, USA). CI < 1 suggests synergy, while CI > 1 indicates antagonism.

## 5. Conclusions

**Figure 8:**
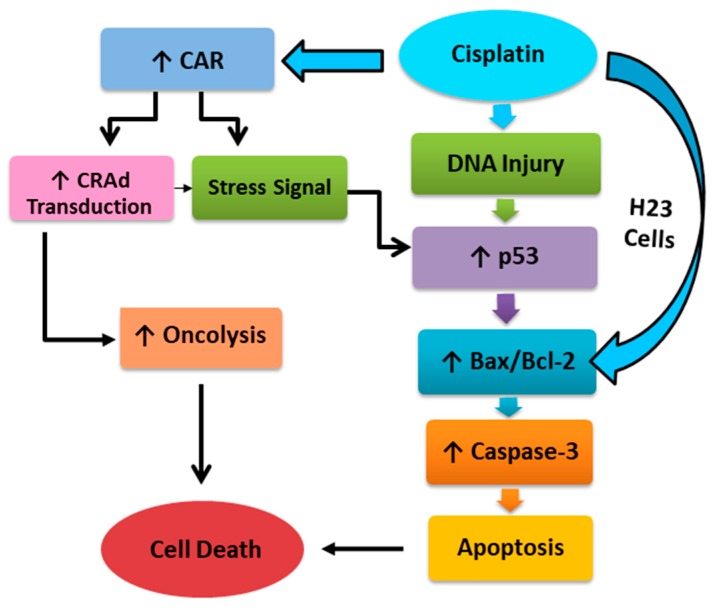
Molecular mechanism underlying cisplatin and CRAd induced cancer cell death.

In conclusion, the cisplatin synergistically increased the tumor-killing of CRAd by (1) increasing CRAd transduction via enhanced CAR expression and (2) increasing p53 dependent or independent apoptosis of lung cancer cell lines (Figure 8). Also, CRAd alone displayed very efficient anti-tumor activity in cancer cells resistant to cisplatin owing to upregulated CAR levels. In an exciting outcome, we have revealed novel therapeutic opportunities to exploit intrinsic and acquired resistance to enhance the therapeutic index of anti-tumor treatment in lung cancer.

## Figures and Tables

**Table 1:** Primer sequences for RT-PCR.

Gene	Primer-F	Primer-R	Ref.
CAR	GCAGGAGCCATTATAGGAACTTTG	GGACCCCAGGGATGAATGAT	[38]
GAPDH	GAAGGTGAAGGTCGGAGTC	GAAGATGGTGATGGGATTTC	[38]
ABCB1 or MDR1	GGAGCCTACTTGGTGGCACATAA	TGGCATAGTCAGGAGCAAATGAAC	[39]
Survivin	AGAACTGGCCCTTCTTG GAGG	CTTTTTATGTTCCTCTAT GGGGTC	[40]
p53	TAACAGTTCCTGCATGGGCGGC	AGGACAGGCACAAACACGCACC	[40]
E-Cadherin	TCACCACTGGGCTGGACCGA	TACAGCCTCCCACGCTGGGG	[41]
N-Cadherin	TCAAACACAGCCACGGCCGT	CGGTCTGGATGGCGAACCGT	[41]
Vimentin	TTCTCTGCCTCTTCCAAACTTT	CGTTGATAACCTGTCCATCTCTA	[42]
ABCG2 (BCRP)	TTCGGCTTGCAACAACTATG	TCCAGACACACCACGGATAA	[43]
ABCC1(MRP1)	CTCTATCTCTCCCGACATGACC	AGCAGACGATCCACAGCAAAA	[44]
ABCC2(MRP2)	CCCTGCTGTTCGATATACCAATC	TCGAGAGAATCCAGAATAGGGAC	[44]

## Abbreviations

CAR	Coxsackievirus and adenovirus receptor
CRAd	Conditionally Replicating Adenovirus
Bcl-2	B-cell lymphoma 2
Bax	Bcl-2-associated X
EMT	Epithelial to mesenchymal transition
CPR	Cisplatin resistant
RT-PCR	Reverse transcription polymerase chain reaction
MTT	(*3*-(*4*,*5*-dimethylthiazol-*2*-yl)-*2*,*5*-diphenyltetrazolium bromide) tetrazolium
MDR1	Multidrug resistance gene 1
MDR	Multidrug resistance
PCR	Polymerase chain reaction
DMSO	Dimethyl sulfoxide
DDPR	Diamminedichloroplatinum resistant
DMEM	Dulbecco’s modified eagle’s medium
FBS	Fetal bovine serum
EMT	Epithelial–mesenchymal transition
RIPA	Radioimmunoprecipitation assay buffer
PVDF	Polyvinylidene difluoride
GAPDH	Glyceraldehyde 3-phosphate dehydrogenase
